# A Double Blind Clinical Trial on the Efficacy of Honey Drop in Vernal Keratoconjunctivitis

**DOI:** 10.1155/2014/287540

**Published:** 2014-02-24

**Authors:** Ali Salehi, Solmaz Jabarzare, Mohammadreza Neurmohamadi, Soleiman Kheiri, Mahmoud Rafieian-Kopaei

**Affiliations:** ^1^Feiz Hospital-Ophthalmology Center, Isfahan University of Medical Sciences, Qods Square, Isfahan, Iran; ^2^Medical Plants Research Center, Shahrekord University of Medical Sciences, Shahrekord, Iran; ^3^Clinical Biochemistry Research Center, Shahrekord University of Medical Sciences, Shahrekord, Iran

## Abstract

*Purpose*. This trial was designed to evaluate the efficacy and safety of topical honey eye drops in patients with diagnosed VKC. *Methods*. This clinical trial was conducted on 60 patients with diagnosed VKC. The patients were selected and randomly allocated between two groups of 30. Patients in two groups received honey eye drop (60% in artificial tear) or placebo, other than cromolyn and fluorometholone 1% eye drops, to be used topically in each eye, four times per day. The patients were examined with slit lamp and torch at baseline and the follow-up visits on the 1st, 3rd, and 6th months of the study for redness, limbal papillae, and intraocular pressure. *Results*. Out of 60 patients who completed the study, 19 patients (31.7%) were female. There was significant increase in eye pressure and reduction in redness as well as limbal papillae, following the consumption of the honey drop in honey group compared to placebo control group (*P* < 0.05). At the end of trial, one patient in honey group and 7 ones in placebo group had limbal papillae (*P* < 0.05). *Conclusion*. Topical honey eye drops, when used along with Cromolyn and Fluorometholone eye drops, might be beneficial for the treatment of VKC.

## 1. Introduction

Vernal keratoconjunctivitis (VKC) is a seasonal recurrent disease which is considered as an inflammatory or allergic ocular disease. It is mostly seen in children and young adults and the incidence of disease is high in spring and summer [1, 2]. Vernal keratoconjunctivitis has been reported mostly in tropical countries such as Pakistan and may cause difficulties for the cornea and conjunctiva [2, 3].

Patients usually complain about severe eye irritation, photophobia, chemosis, tearing, and excessive mucus secretions that lead to stick eyelash edges. Large amounts of IgE and secreted proteins of eosinophils are found in the tears of patients with vernal keratoconjunctivitis [4, 5]. Many fine papillae can be seen in inferior tarsal conjunctiva. There are giant papillae in upper eyelid conjunctiva that create a paving appearance [6].

Therapeutic options for this disease include topical steroids, antihistamines, and mast cell stabilizers. Mast cell stabilizers are preferred because of fewer side effects [7]. The most important and the most serious side effect of steroids is high eye pressure which ultimately may lead to glaucoma arising in patients with genetic predisposition. Other side effects can include cataract and secondary infection. About ten percent of the patients will be inflicted with corneal ulcers which may lead to decreased vision due to corneal changes. Other patients' ocular difficulties may lead to glaucoma, cataract, or large corneal pannus [6].

Recently the use of alternative and complementary medicine has been increasing [8]. During the last decade the number of people who has tried alternative and complementary medicine is nearly doubled and the numbers remain on the rise [9]. Furthermore, promising results have been released for alternative therapy in various kinds of diseases [10, 11].

Honey has been used in the treatment of eye diseases as medicine for thousands of years. Nowadays, more attention is paid to it because of numerous and suitable reports about the properties of honey. It has antibacterial and antifungal effects on a wide range of microorganisms. Furthermore, with regard to the decrease of the efficacy of antibiotics due to resistant strains and resistance to antibiotics which is a serious threat to global health, the efficacy of such honey has been proven effective in improving eye diseases [12]. For instance, it is used in the treatment of corneal bacterial ulcers, catarrhal keratoconjunctivitis, syphilis keratitis, corneal calcareous burns, other keratitis, keratoconjunctivitis, corneal infections, and chemical and thermal eye burns [13]. Due to the side effects of available drugs in keratoconjunctivitis and weak curative effects, this study was designed with the aim of determining the effect of the honey drop on keratoconjunctivitis in order to find out a way to reduce consumption of steroids in these patients.

## 2. Materials and Method

This clinical trial study was done on 60 patients referred to the ophthalmology clinic in Shahrekord, Iran. The patients were examined and selected by an ophthalmologist based on history and clinical examinations. Patients who had mild, moderate, or severe kinds of keratoconjunctivitis, had no other ocular diseases, and had not previously received any treatment for keratoconjunctivitis were included to reduce the impact of potential confounders. They were attended in the study with personal satisfaction. Patients who had other ocular problems or complications were excluded from the study. The sampling method was based on convenience sampling. Patients were randomly allocated between two groups of 30. The sample size was calculated based on a 95% confidence and a power of 80% to see a reduction of redness from 65% to 30% in the case group. The sample size was obtained as 30 patients in each group.

Patients in first group received fluorometholone drop (1%) and sodium cromolyn along with honey drops (60% honey in artificial tears) and patients in second group received fluorometholone and sodium cromolyn along with artificial tear. All patients were asked to use 1 drop from each of the mentioned treatments every 6 hours for 1 month. During the study period, patients were initially examined once a week for the first month of the study and then 3 and 6 months from the first visit. Patients were examined by slit lamp in terms of symptoms such as redness of the eye, limbal papillae, intraocular pressure, and visual acuity.

Throughout the study research the tenets of the Declaration of Helsinki [14] were followed and informed consent was obtained. The research was approved by Ethical Committee and review board of Shahrekord University of Medical Science. Power analysis was performed to justify the number of patients enrolled in the study.

Results were presented based on the percentage for qualitative variables and mean and standard deviation for quantitative variables. Statistical analysis was done using independent and paired *t*-tests and Chi-square or Fisher's exact tests by SPSS. *P* < 0.05 was considered statistically significant.

## 3. Results

In this clinical trial, all 60 patients completed the study in two groups of experimental (*n* = 30) and control (*n* = 30). Nineteen (31.7%) patients were female and the rest were male. The sex difference was not observed between the two groups (*P* < 0.05).

Eye pressure results of patients' left and right eyes in both groups are summarized in Table 1. In group 1, the left and right eye pressure were significantly increased after the study but it was in normal range.

There was a significant difference in the amount of right eye limbal papillae in experimental group compared to control group after study (*P* < 0.05), representing 100% recovery of the right eye. In left eye, only one person from experimental group had limbal papillae after study while 7 ones in control group had limbal papillae. Generally, the amount of limbal papillae in experimental group was significantly decreased during the study (*P* < 0.05) (Table 3).

## 4. Discussion

This study aimed to determine the effect of honey drop on the symptoms of vernal keratoconjunctivitis and it was designed to find out a way to reduce the amount of corticosteroid usage. The results of this study showed that the use of honey drop in the treatment of vernal keratoconjunctivitis caused eye redness to improve, the limbal papillae to reduce, and allergic symptoms to improve. Allergic reactions are created as a result of the severe response to allergic materials.

It has been suggested that the most important participating cells in vernal keratoconjunctivitis are eosinophils. Sometimes, white spots are seen in conjunctiva and cornea due to the accumulation of inflammatory cells. Anti-inflammatory effect of honey on wound tissues has been observed by microscopic studies with reduction in white blood cells, such as eosinophils, involved in inflammation [6].

Topical steroids as primary treatment for swollen eye or vernal keratoconjunctivitis are widely used with remarkable effect in improving symptoms such as intense itching, photophobia, and tearing. However, prolonged use of topical steroids leads to increase of the risk of cataracts; corneal thinning; and fungal, viral, and bacterial infections. All of these conditions can lead to blindness [2].

In this study, using a honey drop (60%) had great impact on the reduction of eye redness and improvement of limbal papillae compared to control group. It has been reported that honey can reduce inflammation, edema, and exudate removal [15]. Considering that vernal keratoconjunctivitis is an allergic inflammatory disease of the eye, possibly honey has been effective in improving symptoms by reducing inflammation. Previous investigations have concluded that honey might be a good remedy for ocular wounds and it is used as a panacea for eye diseases [16, 17].

Honey has antibacterial effect and can be used to prevent corneal scarring caused by infection [17, 18]. Healing of bacterial corneal wound has been reported by honey. Honey can also probably be effective in improving the scars after infections [19, 20]. The anti-inflammatory effect of honey has caused it to be used in the treatment of blepharitis (inflammation of the eyelid margins) and keratitis (corneal inflammation) [21].

Honey has clear antioxidant property and cause free radicals to neutralize. Antioxidant activity could be one reason for the anti-inflammatory effect of honey. Because oxygen free radicals are involved in different inflammatory conditions [22–24], antioxidants, especially the natural ones, are able to reduce inflammation [25–27]. Even if honey antioxidants cannot completely suppress inflammation, it can reduce its damage and possibly be effective in improving the symptoms of vernal keratoconjunctivitis.

In clinical trials like this, the patient compliance is very important. The viscosity of honey drop is more than tear and its usage may cause inconvenience for a few seconds. Although this might have caused a limitation; however, the results show that the majority of patients might have used their medications.

There was significant increase in eye pressure following the consumption of the honey drop in honey group compared to placebo control group (*P* < 0.05). At the end of trial one patient in honey group and 7 ones in placebo group had limbal papillae (*P* < 0.05).

The results showed that drop of honey can be effective in reduction of redness and limbal papillae and in improving the vernal keratoconjunctivitis. It might be used to reduce the amount of steroid consumption. However, more research is needed to determine how much it can reduce the need for steroid usage.

## Figures and Tables

**Table 1:** The mean of eye pressure in two groups.

Involvement eye	Steps	Experimental	Control	Sig.*
Right	Before	13.03 (1.69)	13.27 (2.24)	0.651
	After	14 (1.36)	16.43 (2.20)	0.001
	Sig.**	0.001	0.001	—

Left	Before	13.27 (1.33)	13.10 (2.32)	0.735
	After	14.30 (1.26)	16.77 (2.30)	0.001
	Sig.**	0.001	0.001	—

*Between two groups sig. Independent *t*-test was used.

**Before and after sig. Paired *t*-test was used.

The redness in both left and right eyes was improved in patients of group 1 who received honey drop compared to control group (Table 2).

**Table 2:** Frequency of the intensity of redness in patients' eyes in both groups.

Eye	Step	Redness	Experimental	Control	Sig.
Right	Before study	Normal	9	6	0.165
		Severe	21	21	
		More severe	0	3	
	After study	Without redness	13	0	0.001
		Low	13	1	
		Normal	3	15	
		Severe	1	14	

Left	Before study	Normal	10	5	0.111
		Severe	20	22	
		More severe	0	3	
	After study	Without redness	15	0	0.001
		Low	12	2	
		Normal	3	15	
		Severe	0	13	

**Table 3:** Comparison of limbal papillae in the experimental and control groups.

	Step	Experimental group		Control group		Sig.
		Number	Percent	Number	Percent	
Right eye	Before study	16	53.3	14	46.7	0.60
	After study	0	0	13	43.3	0.001

Left eye	Before study	18	60	11	36.7	0.071
	After study	1	3.33	7	23.3	0.052
